# Diverse Effects of the NTCP p.Ser267Phe Variant on Disease Progression During Chronic HBV Infection and on HBV preS1 Variability

**DOI:** 10.3389/fcimb.2019.00018

**Published:** 2019-03-01

**Authors:** Fangji Yang, Lina Wu, Wenxiong Xu, Ying Liu, Limin Zhen, Gang Ning, Jie Song, Qian Jiao, Yongyuan Zheng, Tongtong Chen, Chan Xie, Liang Peng

**Affiliations:** ^1^Department of Infectious Diseases, The Third Affiliated Hospital of Sun Yat-sen University, Guangzhou, China; ^2^Department of Severe Liver Disease, Guangzhou Eighth People's Hospital, Guangzhou Medical University, Guangzhou, China; ^3^Guangdong Province Key Laboratory of Liver Disease Research, The Third Affiliated Hospital of Sun Yat-sen University, Guangzhou, China

**Keywords:** sodium taurocholate co-transporting polypeptide (NTCP), hepatitis, hepatitis B virus (HBV), preS1 region, single nucleotide polymorphism (SNP)

## Abstract

The sodium taurocholate co-transporting polypeptide (NTCP) acts as a cellular receptor for the hepatitis B virus (HBV) infection on host hepatocytes. We aim to investigate how the NTCP p.Ser267Phe variant affects HBV-related disease progression and analyze viral genomic variability under a host genetic background carrying the p.Ser267Phe variant. A total of 3187 chronic hepatitis B (CHB) patients were enrolled and genotyped for the p.Ser267Phe variant. The variant's association with disease progression was evaluated by logistic regression analysis. We also enrolled 83 treatment-naive CHB patients to analyze the variability of the HBV preS1 region. The frequency of the NTCP p.Ser267Phe variant was significantly lower in patients diagnosed with acute-on-chronic liver failure [OR (95% CI) = 0.33 (0.18–0.58), *P* = 1.34 × 10^−4^], cirrhosis [OR (95% CI) = 0.47 (0.31–0.72), *P* = 4.04 × 10^−4^], and hepatocellular carcinoma [OR (95% CI) = 0.54 (0.34–0.86), *P* = 9.83 × 10^−3^] as compared with CHB controls under the additive model after adjustment. Furthermore, the percentage of amino acid mutations in HBV preS1 region was significantly higher in the NTCP p.Ser267Phe heterozygote group than in the NTCP wild type homozygote group (*P* < 0.05). We herein demonstrate that the NTCP p.Ser267Phe variant is a protective factor reducing CHB patient risk for liver failure, cirrhosis, and hepatocellular carcinoma. A host genetic background carrying NTCP p.Ser267Phe exerts selective pressure on the virus, leading to more variability.

## Introduction

Hepatitis B virus (HBV) infection constitutes a public health challenge, which affects more than 250 million people worldwide (Trepo et al., 2014). Chronic HBV infection confers a higher risk of hepatitis, liver cirrhosis, and hepatocellular carcinoma (HCC) (Lok and McMahon, 2007). Varying clinical outcomes of HBV infection are affected by a complex combination of virus and host factors, such as viral genomic variability, host immunological state, and host genetic background (Hu et al., 2013; Al-Qahtani et al., 2018; Tan et al., 2018b). The mechanism of HBV-related disease progression has not yet been fully elucidated.

The HBV virus comprises an external envelope composed of surface glycoproteins, an icosahedral nucleocapsid, and a 3.2 kb partially double-stranded DNA genome. The three surface proteins [small (S), middle (M), and large (L)] have a common C-terminal of S domain, but the M and L proteins have different N-terminal domain lengths (Heermann et al., 1984; Seeger and Mason, 2000). HBV manifests significant hepatotropism, and only human and *Tupaia* hepatocytes are susceptible to HBV infection. Recently, it was discovered that HBV entry into human hepatocytes is mediated by the receptor sodium taurocholate co-transporting polypeptide (NTCP) expressed by the host (Yan et al., 2012; Ni et al., 2014). The preS1 domain of large envelope proteins is responsible for its binding with NTCP and involved in virus–host receptor interaction (Barrera et al., 2005; Glebe et al., 2005; Yan et al., 2012).

NTCP, encoded by *SLC10A1*, is a sodium–bile acid cotransporter that facilitates their transfer into hepatocytes from portal blood as part of the enterohepatic circulation (Stieger, 2011). Previous studies demonstrated that specific single nucleotide polymorphisms (SNPs) alter the physiological function of NTCP including bile salt homoeostasis, HBV entry, and clinical outcomes of HBV infection (Ho et al., 2004; Peng et al., 2015; Liu et al., 2017). Most *SLC10A1* SNPs have distributions related to ethnicity, and the non-synonymous mutation that encodes the p.Ser267Phe variant (S267F, c.800 G>A, rs2296651) is specific to Asian patient populations (Pan et al., 2011; Lee et al., 2017). Our previous work found an association between the chronic hepatitis B (CHB) resistance and the p.Ser267Phe variant (Peng et al., 2015), and several other studies have come to the same conclusion (Hu et al., 2016; Wang et al., 2017; An et al., 2018). Furthermore, some studies found that the Ser267Phe variant is inversely associated with HBV-related disease progression to cirrhosis and HCC (Hu et al., 2016; An et al., 2018). However, several studies did not observe a protection effect of the minor allele of the p.Ser267Phe variant against HBV infection or HBV-related disease progression (Su et al., 2014; Zhang et al., 2017). We, therefore, performed a genetic association study, enrolling a large cohort of 3,187 chronic HBV infected patients to comprehensively assess the relationship between the NTCP p.Ser267Phe variant and the HBV-related clinical outcomes including cirrhosis, liver failure and HCC.

HBV has been evolving together with *Homo sapiens* over millennia and many studies have reported the evolutionary “arms race” between the HBV and the host immune system (Kramvis et al., 2018; Lumley et al., 2018). Recently, two studies reported that HBV-related hepadnaviruses drive the evolutionary history of their mammalian host receptor and induce signatures of adaptive selection in the cellular receptor NTCP (Jacquet et al., 2018; Takeuchi et al., 2018). Host genes also influence the evolution of the virus. For example, the genetic polymorphisms of the TRIM5 and APOBEC3G genes in primates exert selective pressure on the evolution of simian immunodeficiency virus (Kirmaier et al., 2010; Compton et al., 2012). Previous studies report that the p.Ser267Phe variant of NTCP abolishes or reduces the infection of HBV *in vivo* and *in vitro* (Yan et al., 2014; Peng et al., 2015). Therefore, we hypothesize whether the p.Ser267Phe variant of NTCP exerts selective pressure on HBV to drive virus evolution. Considering that preS1 region of HBV is responsible for its binding with NTCP and that this interaction mediates the early viral entry process (Yan et al., 2012), we, therefore, analyzed the variability and conservation of preS1 region in the presence of the host's p.Ser267Phe variant.

## Patients and Methods

### Cohort and CHB Progression Group Definition

From May 2008 to January 2012, we collected data from 1,899 Han Chinese patients with chronic HBV infection from the Third Affiliated Hospital of Sun Yat-Sen University in Guangzhou, Guangdong Province, China. A total of 1,817 patients from the previous cohort who met the criteria for the present study were included in this study as cohort I, and their clinical data were last updated on April 30, 2018. From January 2016 to April 2018, 1,370 patients with chronic HBV infection were enrolled from the same center as cohort II. A total of 3,187 individuals with chronic HBV infection were enrolled in the project.

Cases in cohorts I and II were consistent with the inclusion and exclusion criteria for chronic HBV infection as in our previous studies (Peng et al., 2015). The diagnosis of chronic HBV infection was based on seropositivity of hepatitis B surface antigen (HBsAg) over 6 months in accordance with the Chinese guideline of prevention and treatment for CHB (Chinese Society of Hepatology and Chinese Society of Infectious Diseases, 2011). Cases who were pregnant, were coinfected with hepatitis A, C, D, E, or human immunodeficiency virus, and were suffering from autoimmune diseases or from alcohol or drug-induced hepatitis or fatty liver disease were excluded. Patients in the CHB control group were screened of laboratory tests (platelet count, international normalized ratio of blood clotting, and alpha-fetoprotein) and medical imaging examinations [hepatic ultrasound, computed Tomography (CT), or magnetic resonance imaging (MRI)] to exclude the presence of cirrhosis, HCC, and liver failure. Cases in the cirrhosis group should be the evidence of decompensation such as image diagnosis of splenomegaly, portal hypertension ascites, hypoproteinemia (<36 g/L), or thrombocytopenia (<1 × 10^9^/L). Patients with liver failure or HCC were excluded from the cirrhosis group. Inclusion criteria for the acute-on-chronic liver failure (ACLF) group were according to consensus recommendations of the Asian Pacific Association for the Study of the Liver (APASL) (Sarin et al., 2009). Hepatic encephalopathy (HE) is defined by the scale provided in the AASLD 2014 Hepatic Encephalopathy Guidelines (AASLD and EASL, 2014), and the HE group only enrolled the overt HE cases. Patients in the HCC group were diagnosed based on confirmed histopathological evidence by two independent pathologists, or at least two imaging examinations (hepatic ultrasound together with CT or MRI) with elevated serum α-fetoprotein level.

This study was carried out in accordance with the guidelines of the 1975 Declaration of Helsinki. Before implementation of the project, approval from the Ethics Committee of the Third Affiliated Hospital of Sun Yat-Sen University was obtained. All subjects were informed and provided written informed consent.

### Clinical Parameters

We collected clinical parameters including serum alanine aminotransferase (ALT), aspartate aminotransferase (AST), HBsAg, hepatitis B e-antigen (HBeAg), and HBV DNA loads from the hospital information system, and the test method was described as previously reported (Zhang et al., 2016). The HBV DNA load was monitored prior to antiviral therapy. Other baseline information was collected during each patient's first clinical examination after disease onset.

### Genomic DNA Extraction, Sequencing, and Genotyping

Genomic DNA was extracted from whole blood using the Blood Genomic DNA Extraction Kit (Tiangen, Beijing, China) according to the manufacturer's instruction. The genomic DNA was stored at −80°C in a freezer until used. Five target SNPs (rs201339654, rs202018997, rs2296651, rs61745930, and rs759531965) in *SLC10A1*, which were reported to affect HBV infection or bile acid transport *in vitro*, were selected for analysis (Ho et al., 2004; Pan et al., 2011; Lou et al., 2014; Fu et al., 2017; Muller et al., 2018). Polymerase chain reactions (PCR) were carried out strictly following a standardized protocol. Information on these five SNPs and their sequencing primers are provided in Tables S1, S2.

We first performed a SNaPshot multiplex assay with the five SNPs identified in 574 DNA samples from cohort II. Apart from rs2296651 G>A, no heterozygous or mutant homozygous individuals of the other four SNPs were found. Therefore, we followed the Sanger sequencing method, consistent with previous studies, to genotype rs2296651 in the remaining 796 samples from cohort II. SNPs were visualized using GeneMapper 4.1 (Applied Biosystems, Foster City, CA, USA).

### HBV Viral DNA Isolation and HBV Sequencing

We extracted HBV DNA from 200 ul of patient serum samples using a QIAamp MinElute Virus Spin Kit (QIAGEN, Hilden, Germany), following procedures provided by the manufacturer. A DNA fragment containing the preS1 region was specifically amplified using Q5 Hot Start High-Fidelity Master Mix (New England Biolabs, MA, USA) with the following primers: forward, 5′-AAGGTGGGAAACTTTACGGG-3′; reverse, 5′-TGACAWACTTTCCAATCAATAGG-3′. PCR conditions are as follows: 98°C for 30 s; 98°C for 10 s, 55°C for 20 s, and 72°C for 50 s for 35 cycles, and 72°C for 2 min (final extension). PCR products were sequenced using the BigDye Terminator v3.1 Cycle Sequencing Kit (Invitrogen, CA, USA), and sequencing was performed on an ABI 3730XL DNA analyzer. The HBV sub-genomic fragments were submitted to the NCBI website to identify the genotype (http://www.ncbi.nlm.nih.gov/projects/genotyping/formpage.cgi). We aligned and compared the amplified HBV sequences with the HBV genotype B (GenBank accession no. D00330) or genotype C (GenBank accession no. AB033556) reference sequence to determine preS1 fragment variants.

### Data Analysis

Statistical analysis of SNP associations was performed using PLINK software (Purcell et al., 2007). Variation associated with diagnosis was tested using logistic modeling with correction for gender, age, lg HBV DNA, AST, ALT, and HBeAg status. The codominant, dominant, recessive, and additive genetic models were applied to calculate the genotype frequencies. A Bonferroni- adjusted *P* value < 0.05 was considered statistically significant. Odds ratios (OR) and 95% confidence intervals (95% CI) were also calculated. Linear regression modeling of SNP genotype and lg HBV DNA, AST, ALT, and HBeAg status was applied to estimate the correlation, with correction for diagnosis. Deviation from the Hardy–Weinberg equilibrium (HWE), expectation for any genetic variant was analyzed using the chi-square test. The distributions of point variations at the amino acid level between groups were analyzed using Fisher's exact test. Amino acid mutation sites appearing in more than two sequences were included in the analysis. Between-group differences in qualitative data (gender, HBeAg status) were assessed using chi-square tests, and the quantitative data (age and lgHBV) were assessed using Student's *t*-tests. A two-tailed *P* value < 0.05 was considered statistically significant.

## Results

### Demographic and Clinical Characteristics

A total of 3,187 participants, including CHB controls (*n* = 1,426), individuals with ACLF (*n* = 535), individuals with cirrhosis (*n* = 753), individuals with HCC (*n* = 473), were enrolled in the study. The demographic and clinical characteristics of the recruited population are summarized in Table 1. Participants had a mean age of 44.22 years and comprised 2,632 men and 555 women. Mean age of each group increased in the order of CHB (35.56 ± 10), ACLF (41.61 ± 10), Cirrhosis (CIR) (46.67 ± 10), and HCC (47.82 ± 10). The genotypes of the p.Ser267Phe variant in the CHB group, CIR group, HCC group, and all participants combined were distributed in line with HWE (*P* > 0.05). In ACLF group, the allele frequency of the p.Ser267Phe variant was lower than expected and did not conform to HWE (*P* = 0.017).

**Table 1:** Demographic and clinical features in the study groups.

**Characteristic**	**ALL subjects**	CHB^*^ controls	**ACLF^§^ group**	**CIR^‡^ group**	**HCC^†^ group**
Cohort I	1,817	967	223	334	293
cohort II	1,370	459	312	419	180
Cohort I and Cohort II	3,187	1,426	535	753	473
Gender, Male (%)	2,632 (82.5%)	1,105 (77.49%)	473 (88.41%)	618 (82.07%)	436 (92.18%)
Age, year, mean ± SD	44.22 ± 11	35.56 ± 10	41.61 ± 10	46.67 ± 10	47.82 ± 10
HBV DNA (lgIU/mL), mean ± SD	5.31 ± 2.21	5.90 ± 2.10	5.38 ± 2.15	4.47 ± 2.27	4.65 ± 1.91
ALT (IU/L), median (quartile)	78 (38–358)	74 (36–362)	400 (91–1177)	56 (33–136)	50 (30–92)
AST (IU/L), median (quartile)	83 (36–271)	56 (23–222)	403 (138–1029)	70 (39–167)	70 (38–139)
HBeAg positive (%)	1417 (44.46%)	833 (58.42%)	178 (33.27%)	258 (34.26%)	148 (31.29%)

*ALT, alanine aminotransferase; AST, aspertate aminotransferase; HBeAg, hepatitis B e antigen*.

* *Patients have chronic hepatitis B without liver cirrhosis, hepatocellular carcinoma or liver failure*.

† *Patients have hepatitis B virus related-hepatocellular carcinoma*.

‡ *Patients have hepatitis B virus related-liver cirrhosis*.

§ *Patients have hepatitis B virus related-acute-on-chronic liver failure*.

### The NTCP p.Ser267Phe Variant and Risk for HBV- Related ACLF

To assess the association between the p.Ser267Phe variant and the HBV-related ACLF, patients with ACLF were compared with CHB controls (Table 2). The minor allele frequency (MAF) of the p.Ser267Phe variant in the ACLF group was significantly lower than that in the CHB controls [OR (95% CI) = 0.31 (0.20–0.49); *P* = 6.59 × 10^−8^]. Under the dominant model, the frequency of GA+AA genotypes in ACLF patients was significantly lower than that in the CHB controls [OR (95% CI) = 0.30 (0.16–0.54); *P* = 6.05 × 10^−5^]. In the additive model, the estimated allelic OR is 0.44 (*P* = 7.67 × 10^−7^). Genetic analysis demonstrated that the p.Ser267Phe variant is inversely correlated with HBV-related ACLF, a result that is consistent with our previous work.

**Table 2:** NTCP p.Ser267Phe variant and risk for acute-on-chronic liver failure (ACLF).

**Model**	**Genotype**	**CHB controls**	**ACLF group**	**OR (95% CI)**	***P*-value**
Allele	G	2,671 (93.65)	1,048 (97.94)	0.31 (0.20–0.49)	**6.59** **×** **10**^**−8**^
	A	181 (6.35)	22 (2.06)		
Codominant	G/G	1248 (87.52)	515 (96.26)	–	**2.07** **×** **10**^**−4**^
	G/A	175 (12.27)	18 (3.36)	0.28 (0.15–0.51)	
	A/A	3 (0.21)	2 (0.37)	1.56 (0.12–19.97)	
Dominant	G/G	1,248 (87.52)	515 (96.26)	0.30 (0.16–0.54)	**6.05** **×** **10**^**−5**^
	G/A+A/A	178 (12.48)	20 (3.74)		
Recessive	G/G+G/A	1,423 (99.79)	533 (99.63)	1.68 (0.13–21.28)	0.69
	A/A	3 (0.21)	2 (0.37)		
Additive	–	–	–	0.33 (0.18–0.58)	**1.34** **×** **10**^**−4**^

*Data were presented as number (percentage). The differences of allege frequencies were analyzed using chi-square test and genotype frequencies among groups were analyzed using logistic regression models (codominant, recessive, dominant, additive). ORs (Bofferoni adjusted odds ratio) were calculated and reported within the 95% CI (confidence interval). Significant p-values (p < 0.05) are thickened in bold*.

HE is a complex neuropsychiatric syndrome that occurs during liver failure (Swaminathan et al., 2018). Recent study has shown that HE is associated with increased blood levels of ammonia and bile acids, especially the conjugated bile acids (Xie et al., 2018). NTCP plays an important role in maintaining homeostasis enterohepatic circulation of bile acids (Hagenbuch and Meier, 1994; Kullak-Ublick et al., 2004). In order to determine whether HE is related to the p.Ser267Phe variant genotype, we further compared 204 patients with overt HE with 332 ACLF patients without HE. In the additive model, the estimated allelic OR is 2.50 with a *P* value of 0.038, suggesting that NTCP p.Ser267Phe is associated with increased risk of HE in liver failure patients (Table S3).

### The Association Between NTCP p.Ser267Phe Variant and Development of Cirrhosis and HCC

Next, we examined whether the presence of the p.Ser267Phe variant affects the development of HBV-related cirrhosis and HCC. Genetic analysis demonstrated that the p.Ser267Phe variant protects CHB patients from HBV-induced cirrhosis progression regardless of whether the model was codominant (OR = 0.46; *P* = 1.79 × 10^−3^), dominant (OR = 0.46; *P* = 3.81 × 10^−4^), or additive models (OR = 0.47; *P* = 4.04 × 10^−4^; Table 3).

**Table 3:** NTCP p.Ser267Phe variant and risk for cirrhosis (CIR).

**Model**	**Genotype**	**CHB controls**	**CIR group**	**OR (95% CI)**	***P*-value**
Allele	G	2,671 (93.65)	1,457 (96.75)	0.50 (0.36–0.68)	**1.41** **×** **10**^**−5**^
	A	181 (6.35)	49 (3.25)		
Codominant	G/G	1,248 (87.52)	705 (93.63)	–	**1.79** **×** **10**^**−3**^
	G/A	175 (12.27)	47 (6.24)	0.46 (0.30–0.71)	
	A/A	3 (0.21)	1 (0.13)	0.39 (0.03–5.85)	
Dominant	G/G	1,248 (87.52)	705 (93.63)	0.46 (0.30–0.71)	**3.81** **×** **10**^**−4**^
	G/A+A/A	178 (12.48)	48 (6.37)		
Recessive	G/G+G/A	1,423 (99.79)	752 (99.87)	0.42 (0.03–6.30)	0.53
	A/A	3 (0.21)	1 (0.13)		
Additive	–	–	–	0.47 (0.31–0.72)	**4.04** **×** **10**^**−4**^

*Significant p-values (p < 0.05) are thickened in bold*.

Since HCC always occurs on the foundation of cirrhosis (Affo et al., 2017), we assessed the association between the NTCP p.Ser267Phe variant and the HCC. Firstly, we compared HCC patients with CHB controls. The results of the dominant (OR = 0.55; *P* = 0.01) and additive models (OR = 0.54; *P* = 9.83 × 10^−3^) suggested a 45% reduction in risk for developing HCC (Table 4). Secondly, to determine whether the p.Ser267Phe variant affects risk of developing HCC in individuals with cirrhosis, we compared cirrhosis patients with and without HCC. The results of allele frequency (*P* = 0.93), dominant model (*P* = 0.77), and additive model (*P* = 0.86) did not show statistically significant differences (Table S4). This suggests that the p.Ser267Phe variant does not reduce the risk of cirrhosis developing into HCC.

**Table 4:** NTCP p.Ser267Phe variant and development of hepatocellular carcinoma (HCC).

**Model**	**Genotype**	**CHB controls**	**HCC group**	**OR (95% CI)**	***P*-value**
Allele	G	2,671 (93.65)	910 (96.19)	0.58 (0.41–0.84)	**3.53** **×** **10**^**−3**^
	A	181 (6.35)	36 (3.81)		
Codominant	G/G	1,248 (87.52)	437 (92.39)	–	NA
	G/A	175 (12.27)	36 (7.61)	NA	
	A/A	3 (0.21)	0 (0.00)		
Dominant	G/G	1,248 (87.52)	437 (92.39)	0.55 (0.35–0.88)	**0.01**
	G/A+A/A	178 (12.48)	36 (7.61)		
Recessive	G/G+G/A	1,423 (99.79)	473 (100)	NA	NA
	A/A	3 (0.21)	0 (0.00)		
Additive	–	–	–	0.54 (0.34–0.86)	**9.83** **×** **10**^**−3**^

*NA, data not accessible. Significant p-values (p < 0.05) are thickened in bold*.

### The NTCP p.Ser267Phe Variant and Overall Disease Progression

We sought to determine whether the presence of the p.Ser267Phe mutation might affect the overall disease progression in CHB patients. We combined the ACLF, cirrhosis, and HCC groups together as the overall disease progression group and then compared it with the CHB group. The frequency of the minor A allele of the p.Ser267Phe variant in the overall disease progression group was significantly lower than that in CHB in control group [OR (95% CI) = 0.46 (0.36–0.60); *P* = 2.56 × 10^−10^] (Table 5). The estimated ORs of individuals with HBV-related disease progression and CHB controls who are heterozygous (GA) and minor allele homozygous (AA) relative to that of wild type homozygotes (GG) were 0.43 under the dominant model, indicating a protective effect of the A allele against disease progression. However, the recessive model failed to produce a significant difference, probably because only six homozygous AA carriers were enrolled in the study. Overall, we found that the minor allele (A) plays a protective effect in CHB patients, conferring an approximately 70% reduction in risk for developing liver failure (Table 2), a 50% reduction in risk for developing cirrhosis (Table 3), and a 40% reduction in risk for developing HCC (Table 4).

**Table 5:** NTCP p.Ser267Phe variant and overall disease progression.

**Model**	**Genotype**	**CHB controls**	**ACLF and CIR and HCC group**	**OR (95% CI)**	***P*-value**
Allele	G	2,671 (93.65)	3,415 (96.96)	0.46 (0.36–0.60)	**2.56** **×** **10**^**−10**^
	A	181 (6.35)	107 (3.04)		
Codominant	G/G	1,248 (87.52)	1,657 (94.09)	–	**2.82** **×** **10**^**−6**^
	G/A	175 (12.27)	101 (5.74)	0.42 (0.30–0.59)	
	A/A	3 (0.21)	3 (0.17)	0.72 (0.06–8.23)	
Dominant	G/G	1,248 (87.52)	1,657 (94.09)	0.43 (0.31–0.59)	**4.66** **×** **10**^**−7**^
	G/A+A/A	178 (12.48)	104 (5.91)		
Recessive	G/G+G/A	1,423 (99.79)	1,758 (99.83)	0.77 (0.069–8.70)	0.84
	A/A	3 (0.21)	3 (0.17)		
Additive	–	–	–	0.44 (0.32–0.61)	**7.67** **×** **10**^**−7**^

*Significant p-values (p < 0.05) are thickened in bold*.

### NTCP p.Ser267Phe Variant and Clinical Parameters

Additionally, we tested whether the p.Ser267Phe variant had an impact on clinical parameters including HBeAg state and levels of HBV DNA, ALT, and AST. Under an additive model, the p.Ser267Phe variant is inversely associated with ALT and AST levels (β = −114.6, *P* = 1.55 × 10^−4^ and β = −129.4, *P* = 3.14 × 10^−4^, respectively). However, HBV DNA levels and HBeAg status showed no significant differences (Table S5).

### Host Genetic Background of NTCP p.Ser267Phe Variant and HBV preS1 Variability

**Figure 1:**
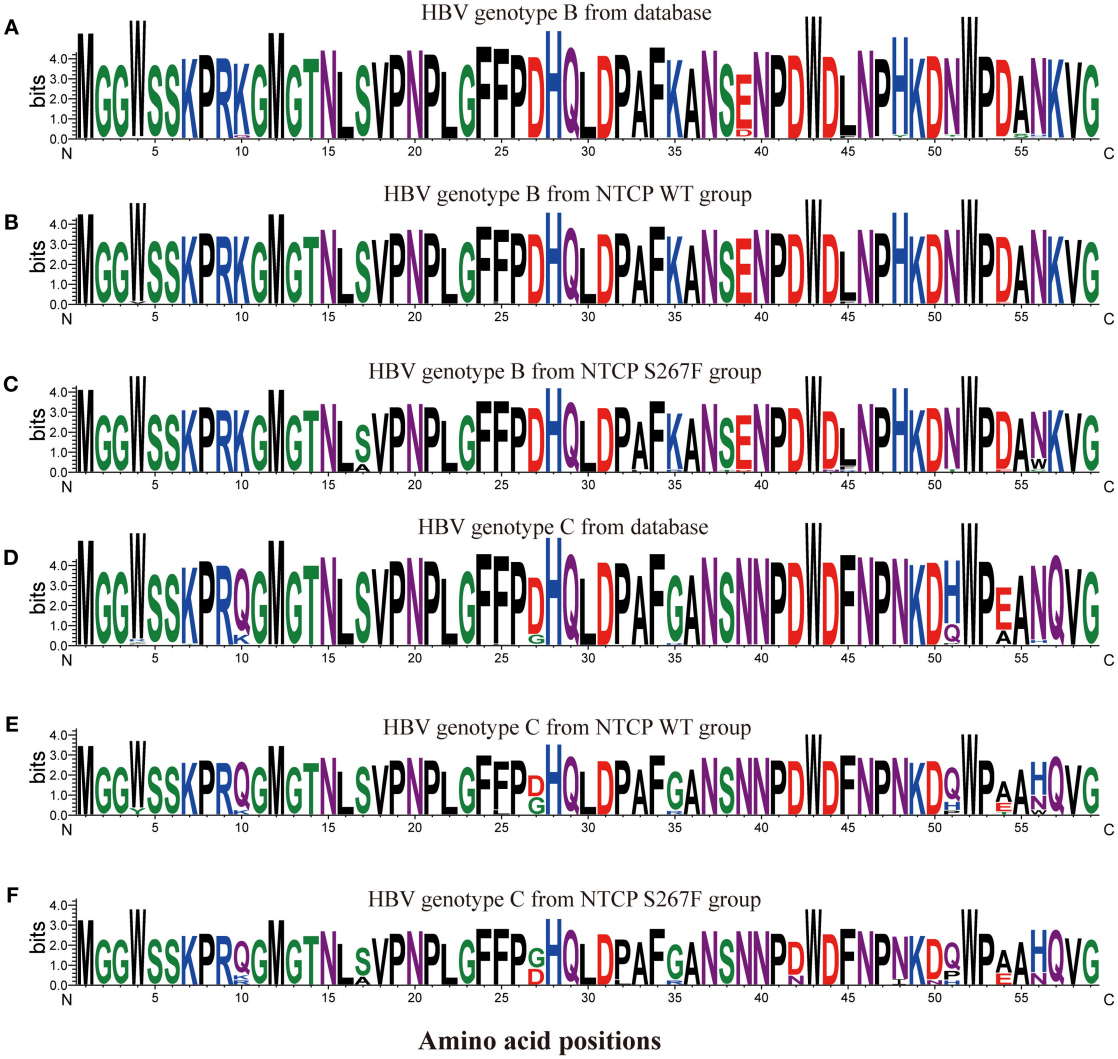
Sequence logos of preS1 amino acids 1–59 from multiple HBV sequences. HBV preS1 amino acids 1–59 sequences were aligned, and the alignment was visualized using the WebLogo tool (http://weblogo.threeplusone.com/). The sequence logos represent calculation of the information content of each amino acid position. 2520 genotype B HBV sequences **(A)** and 2600 genotype C HBV sequences **(D)** were obtained from an online HBV database (https://hbvdb.ibcp.fr/HBVdb/). Thirty-nine sequences **(B)** from the NTCP WT group and 24 sequences **(C)** from the NTCP S267F group were HBV genotype B. 11 sequences **(E)** from the NTCP WT group and 9 sequences **(F)** from the NTCP S267F group were HBV genotype C.

To determine whether the *SLC10A1* genotype exerts selective pressure on HBV, we analyzed the NTCP-interacting domain of the HBV preS1 region. First, we aligned and compared preS1 amino acids (AAs) 1-59 of 2520 genotype B and of 2600 genotype C HBV sequences from an online HBV database (https://hbvdb.ibcp.fr/HBVdb/HBVdbIndex) (Hayer et al., 2013). The results indicate that the preS1 regions of AAs 1-59 in HBV genotype B and C are conserved (Figure 1). Overall preS1 AA composition was visualized using the WebLogo tool (http://weblogo.threeplusone.com/) (Crooks et al., 2004). Next, 83 treatment-naive CHB patients with a viral load >4 lgIU/mL were enrolled in HBV sequence analysis, of which 50 were NTCP wild type homozygotes and 33 were NTCP S267F heterozygotes. The demographic and clinical features of these 83 patients are provided in the Table S6. There are 6 individuals with homozygous p.Ser267Phe mutation in the cohort, and the demographic and clinical features are provided in the Table S7. We can access only the serum from one individual whose HBV DNA viral load is 112 IU/mL, since the other 3 homozygotes are negative for HBV DNA viral load while another 2 homozygotes are lost follow up. The HBV sequences from individuals with homozygous p.Ser267Phe mutation are not included in this study. PreS1 AA 1-59 sequences were aligned and visualized according to the HBV genotype and the NTCP polymorphism status (Figure 1). The view conservation of amino acid sequences was displayed in Figure S1. Four (16.67%) out of 24 genotype B patients with heterozygous S267F mutation harbor the S17A mutation in the preS1 domain, whereas the S17A mutation was not observed in the 39 genotype B patients with wild type NTCP. The percentages of amino acid mutations at positions 17 (which change Ser to Ala) and 56 (which change Asn to Trp) of HBV genotype B were significantly different between the NTCP wild type homozygote and the NTCP S267F heterozygote groups (*P* = 0.0178 and 0.0178, respectively; Table 6). Two (22.22%) out of 9 genotype C patients with heterozygous S267F mutation harbor the S17A and D47N mutations in the preS1 domain, whereas the S17A and D47N mutations were not observed in the 11 genotype C patients with wild type NTCP. The percentages of amino acid mutations at positions 17 and 42 of HBV genotype C were higher in the NTCP S267F heterozygote group than in the NTCP wild type homozygote group, although these differences were not statistically significant (Table 6). The pooled mutation frequencies of AAs 1-59 in both HBV genotypes B and C are statistically significant higher in the NTCP S267F heterozygote group than in the NTCP wild type homozygote group (1.48 vs. 0.22%, *P* < 0.001 and 1.89 vs. 0.62%, *P* = 0.045, respectively). These data indicate that host genetic status at NTCP p.Ser267Phe may exert selective pressure on the HBV virus to drive mutation of the preS1 region.

**Table 6:** Frequency of amino acid changes in the NTCP-interacting domain of HBV preS1 region.

**AA position**	**AA change**	**Genotype**	**NTCP WT group (Mu/all; %changes)**	**NTCP S267F group (Mu/all; %changes)**	***P*-value**
17	S>A	B	0/39, 0	4/24, 16.67	**0.0178**
45	L>F	B	2/39, 5.13	2/24, 8.33	0.6318
56	N>W	B	0/39, 0	4/24, 16.67	**0.0178**
56	N>C	B	0/39, 0	1/24, 4.17	0.3810
17	S>A	C	0/11, 0	2/9, 22.22	0.1895
42	D>N	C	0/11, 0	2/9, 22.22	0.1895

*NTCP WT group, NTCP wild type homozygotes group; NTCP S267F group, NTCP S267F heterozygotes group; mu, mutant. Significant p-values (p < 0.05) are thickened in bold*.

## Discussion

NTCP has been well-established for playing an important role in the enterohepatic circulation of bile acids (Hagenbuch and Meier, 1994; Kullak-Ublick et al., 2004). In 2012, it was identified as a functional cell surface receptor enabling HBV and HDV entries into human hepatocytes in *in vitro* studies (Yan et al., 2012). Genetic polymorphisms in *SLC10A1* have been found to affect NTCP protein function, and some mutations are believed to be specific to certain ethnic backgrounds (Pan et al., 2011). In this study, we screened five SNPs (rs201339654, rs202018997, rs2296651, rs61745930, and rs759531965) in the NTCP gene that have been reported to alter its function *in vitro* (Ho et al., 2004; Pan et al., 2011; Lou et al., 2014; Fu et al., 2017; Muller et al., 2018). This represents the first demonstration that four of these SNPs are all wild type in 574 Han Chinese patients with CHB. The MAF rate of NTCP p.Ser267Phe variant is 4.52% (288/6374) in Han Chinese patients with CHB in our cohort, and the result is consistent with a previous study showing that the p.Ser267Phe mutation is specific to individuals of Asian descent (Pan et al., 2011). Our previous study has found that this mutation site is associated with resistance to CHB in a Han Chinese cohort of 1,899 CHB patients and 1,828 healthy controls (Peng et al., 2015), which is consistent with studies from Taiwan by Hu et al. (2016) and from Korea by Lee et al. (2017).

In this large cohort study enrolling 3,187 CHB patients, we confirmed the NCTP p.Ser267Phe variant is inversely associated with HBV-related disease progression. We found that the minor allele of p.Ser267Phe variant plays a protective effect in CHB patients conferring an approximately 70% reduction in risk for developing liver failure, 50% reduction in risk for developing cirrhosis, and 40% reduction in risk for developing HCC. Our conclusions are generally consistent with those of previous large cohort studies. Hu et al. reported that p.Ser267Phe mutation is inversely associated with progression to cirrhosis and HCC by comparing patients with cirrhosis or cirrhosis-related HCC to those with neither cirrhosis nor HCC in a community-based CHB cohort (Hu et al., 2016). An et al. reported that the NTCP p.Ser267Phe variant is associated with decreased risk for developing HBV-related cirrhosis and HCC in 1,117 Han Chinese patients with HBV infection (An et al., 2018). Admittedly, we found some inconsistent results from other studies, which may be due to differences in numbers of cases enrolled. Lee et al. did not find that the p.Ser267Phe variant is associated with HCC development in a cohort of 1,376 CHB Korean patients (Lee et al., 2017). Wang et al. did not observe any protective effect of the p.Ser267Phe variant when comparing 117 HCC patients with 866 CHB patients but reported an inverse association between this variant and the HCC when performing a systematic review and meta-analysis including 956 HCC patients and 3,759 CHB patients (Wang et al., 2017). In summary, our findings strongly support a beneficial role for the p.Ser267Phe variant.

The mechanism of the protective effect conferred by the p.Ser267Phe variant has not yet been elucidated. We hypothesize that it may be through reduced HBV infection of hepatocytes and bile acid circulation. Yan et al. (2014) and Liu et al. (2018) reported that the p.Ser267Phe mutation renders substantial loss of function or inefficiency in the ability of NTCP to support HBV infection *in vitro*. Our previous work demonstrated that the p.Ser267Phe mutation lies within the channel domain for ligand translocation using a structural model (Peng et al., 2015). This mutation may, therefore, reduce HBV transmission and decrease liver inflammation by interfering with NTCP binding to HBV. Regarding bile acids, these are thought to be cytotoxic, and previous studies have suggested that excessive bile acid concentrations can induce hepatocyte injury by activating the death receptor pathway (Faubion et al., 1999). Ho et al. found that the p.Ser267Phe variant of NTCP produces a near-complete loss of function for bile acid uptake from portal circulation into hepatocytes (Ho et al., 2004). This indicates the possibility of reduced intrahepatic cytotoxic bile acid accumulation and a lower likelihood of hepatic inflammation or hepatocyte injury, resulting in a lower risk of HBV-related cirrhosis, HCC, and ACLF disease progression. In our previous work, we found that homozygosity for p.Ser267Phe in *SLC10A1* is associated with increased serum bile acid levels and reduced vitamin D levels, which indicates that the bile acid enterohepatic circulation is disturbed and that bile acids in the intestine are insufficient (Liu et al., 2017). Recently, Ma et al. found that bile acid metabolism is mediated by gut microbiome and regulates liver cancer via antitumor immunosurveillance by natural killer T cells38 (Ma et al., 2018). In the present study, we also found that the p.Ser267Phe variant is inversely associated with HE among liver failure patients, which indicates that this mutation might affect the enterohepatic circulation of bile acids or the gut microbiome. However, the association between the NTCP p.Ser267Phe variant and the gut microbiome is not yet clear and requires further study.

Since the p.Ser267Phe mutation is associated with HBV infection and CHB disease progression, whether this mutation exerts selection pressure for viral adaption to interact with the cellular receptor of NTCP needs to be examined. HBV is considered to bind NTCP through the N-terminal pre-S domain of the HBV large envelope protein, and the preS1 domain from Gly-2 to Val-47 (based on HBV genotype D, which correspond to Gly-13 to Val-58 for HBV genotypes B and C) of the N-terminus is regarded as the NTCP binding site (Yan et al., 2012). Two previous studies that enrolled 18 patients from Spain and 12 from Korea reported that the NTCP-interacting domain of the HBV preS1 region shows a high degree of conservation, especially in essential residues for NTCP binding (Lee et al., 2017; Casillas et al., 2018). However, our study found more AA mutation positions, such as S17A, D42N, and N56W (based on HBV genotypes B and C) in the preS1 region among NTCP S267F heterozygotes. It appears that a host genotype encoding NTCP p.Ser267Phe exerts selective pressure on the HBV virus to drive mutation of the preS1 region. The possible mechanism underlying the association between the NTCP S267F mutation and higher preS1 (aa 1-59) mutation rate is still unknown. We hypothesized that the possible pathway may be through bile acid transportation. Verrier et al. reported that bile acid, transported through NTCP, modulates the expression of interferon-stimulated genes to affect HCV Infection (Verrier et al., 2016). When the bile acid transportation by NTCP is inhibited by PreS1 peptides, both the Huh7.5.1-NTCP cells and primary human hepatocytes stimulate the expression of genes involved in the IFN-a response. Previous studies have shown that the p.Ser267Phe mutation abolishes most of its function of bile acid uptake *in vitro* experiments (Ho et al., 2004; Yan et al., 2014). Several mutation homozygous cases also demonstrate that the presence of NTCP p.Ser267Phe variant is associated with specific changes in circulating bile acids (Deng et al., 2016; Liu et al., 2017; Tan et al., 2018a). The NTCP S267F mutation may regulate HBV infection by modulating the interferon signaling, which is dependent on its function of bile acid transportation and independent of its receptor function. Unlike the *in vitro* experiment, our data showed that 6 HBV-infected patients (3 for CHB, 2 for ACLF, and 1 for cirrhosis) were homozygotes for NTCP S267F mutation. The NTCP p.Ser267Phe mutation cannot completely block HBV infection *in vivo* for 8.57% (15/175) of p.Ser267Phe mutation homozygotes were reported to be HBV-infected. In detail, 6 of 32 homozygotes in our cohort, 5 of 42 homozygotes in Hu's study (Hu et al., 2016), 2 of 2 homozygotes in Wang's study (Wang et al., 2017), 1 of 2 homozygotes in An's study (An et al., 2018), 1 of 12 homozygotes in Zhang's study (Zhang et al., 2017), and 0 of 85 homozygotes in Nfor's study (Nfor et al., 2018) were reported to be HBV infection. The HBV-infected p.Ser267Phe mutation homozygotes all had low or negative viral loads. A recent study reported that the HBV strains isolated from a CHB patient homozygous for NTCP S267F exhibit two mutations, S17A and D27E, in the preS1 region (Liu et al., 2018). The authors also demonstrate that the HBV with S17A and D27E mutation uses wild type NTCP as a cellular receptor, and the NTCP p.Ser267Phe variant supports HBV infection with a low efficiency. From these data, it seems likely that mutations in the NTCP contact site in the preS1 domain of the large envelope protein may compensate for the NTCP S267F mutation. Whether mutation of preS1 caused by NTCP polymorphisms leads to higher binding affinity with the NTCP protein needs to be further studied, which may be important to optimize hepatitis B and D virus entry inhibitor drugs.

In the present study, we conducted a comprehensive evaluation of genetic associations of the NTCP p.Ser267Phe variant with CHB disease progression. Meanwhile, we evaluated whether the mutation inflicts evolutionary pressure on the NTCP-interacting domain of the HBV preS1 region. However, there are some limitations to our study. First, the distribution of the minor allele was not in accordance with the Hardy–Weinberg equilibrium (*p* < 0.001) in the ACLF group, and we did not perform additional population stratification. Second, we found the protective association between the minor allele GA or AA genotypes with HBV-related disease progression. However, our data also indicated that the minor allele was not associated with the HBV DNA levels and HBeAg status aside from the ALT and AST levels. Our study did not provide additional evidence to address the question how does the p.Ser267Phe variation in NTCP alter the HBV-related clinical outcomes. More experiments and evidence are needed to elucidate the role of NTCP and the p.Ser267Phe mutation in HBV-related disease progression. Third, we only analyzed the preS1 region rather than the whole genome of the HBV virus by performing Sanger sequencing. As previously discussed, the preS1 region of HBV is responsible for binding with NTCP, and if there is a selective pressure exerted by the p.Ser267Phe variant, then the preS1 domain is most likely to evolve adaptive selection to compensate for the mutated NTCP. Our data indicated there are more mutations in the HBV preS1 domain in the NTCP p.Ser267Phe heterozygote. Whether these HBV mutations caused by the p.Ser267Phe variant are specifically observed in the preS1 region or are also seen in other fragments need to be further studied. The percentage of wild type and mutation in HBV sequences from “one patient” and the low-frequency mutations were also inaccessible in our data because Sanger sequencing is not precisely quantifiable and is unable to detect alleles below a threshold of 15–20% (Rohlin et al., 2009). In order to address these question, deep sequencing of the HBV preS1 domain is currently underway. After comprehensive analysis of the adaptive evolution of HBV under host genetic background carrying NTCP p.Ser267Phe, it is necessary to demonstrate whether the adaptive variant affects HBV infection *in vitro* cell culture experiments using HepG2-NTCP cells. It may be interesting to compare the blocking capacities of synthetic preS1 peptide with mutation sites and wild type peptide in inhibiting HBV or HDV entry. At the same time, experiments are needed to assess whether the attachment of preS1 peptide with mutation sites is more robust than the wild type peptide in binding with NTCP protein.

In summary, by assessing all the HBV-related disease progressionas in a large cohort of Chinese CHB patients, the NTCP p.Ser267Phe variant was demonstrated to be protective, reducing the risk of liver cirrhosis, hepatocellular carcinoma, and liver failure in CHB patients. The host genotype encoding NTCP p.Ser267Phe exerts selective pressure on the HBV virus to drive further mutations in the HBV preS1 region. Together with other important risk factors, genetic variation in NCTP and HBV preS1 variability can likely be incorporated into cirrhosis, HCC, and ACLF prediction models to help identify individuals with higher or lower risk of CHB-related advanced liver diseases in Asian areas of endemic HBV infection.

## Author Contributions

LP and CX conceived and designed the study. FY, LW, WX, YL, LZ, and GN recruited the cohort and data. JS, QJ, YZ, and TC extracted the DNA and sequenced the genotype. FY and LW analyzed the data and wrote the manuscript. LP and CX critically revised the manuscript. All authors reviewed the manuscript.

### Conflict of Interest Statement

The authors declare that the research was conducted in the absence of any commercial or financial relationships that could be construed as a potential conflict of interest.
