# Supplementary material for: Diverse Effects of the NTCP p.Ser267Phe Variant on Disease Progression During Chronic HBV Infection and on HBV preS1 Variability
Source: Front Cell Infect Microbiol. 2019 Mar 1;9:18. doi: 10.3389/fcimb.2019.00018 (PMC6407604; doi:10.3389/fcimb.2019.00018)
Supplement: Supplementary file 1 [file Table_1.docx]

**Title: Diverse Effects of The NTCP p.Ser267Phe Variant on Disease Progression During Chronic HBV Infection and on HBV preS1 Variability**

**SUPPLEMENTARY MATERIALS**


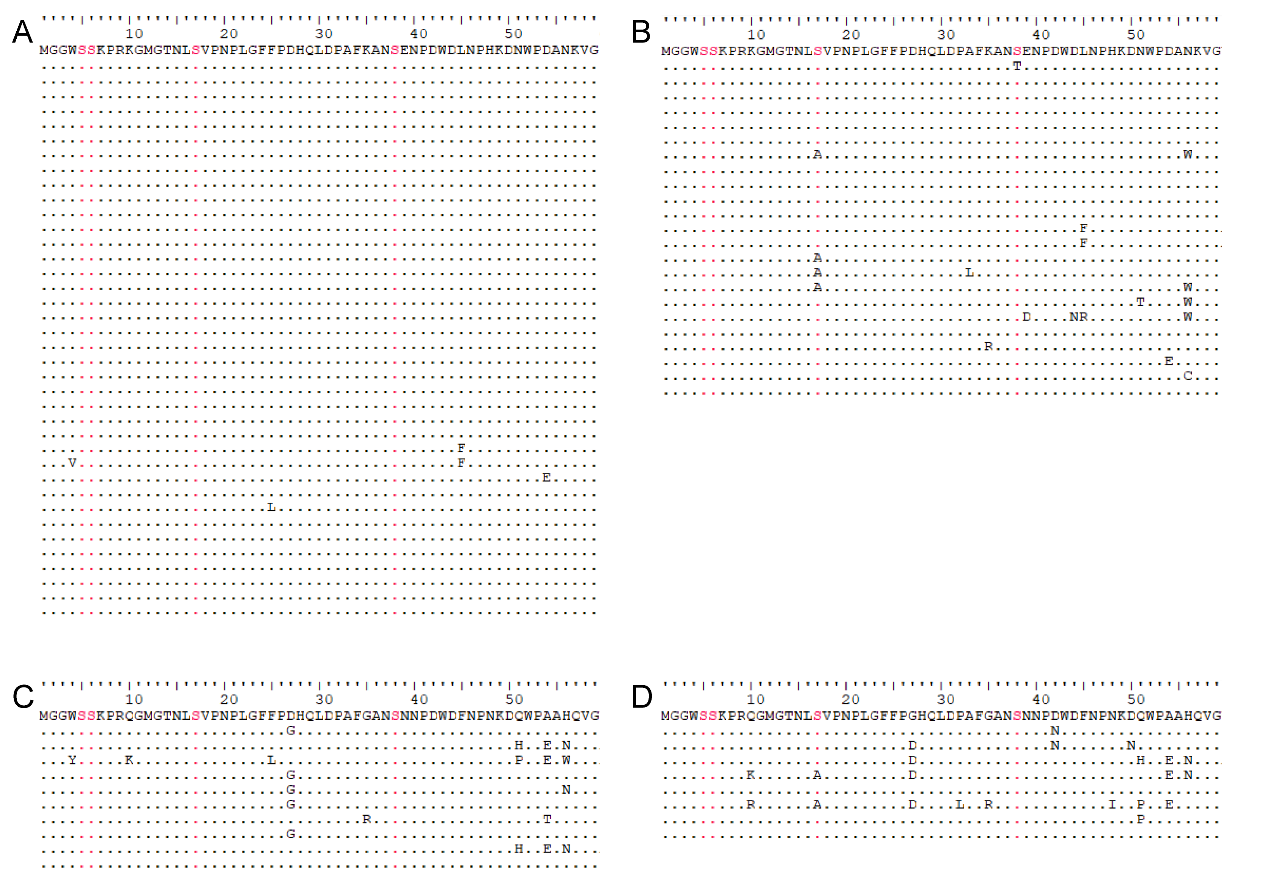


**Supplementary Figure1** PreS1 domain of 1–59 amino acid sequence alignment.

HBV PreS1 amino acid sequences were obtained from the NTCP WT group and NTCP S267F group. The view conservation of amino acid sequences was displayed by plotting identities to a dominant amino acid as a dot. 39 sequences (A) from the NTCP WT group and 24 sequences (B) from the NTCP S267F group were HBV genotype B. 11 sequences (C) from the NTCP WT group and 9 sequences (D) from the NTCP S267F group were HBV genotype C.

Table S1. 5 target SNPs locus in human NTCP

| **SNP ID** | **Region** | **Allele change** | **AA change** | **MAF†** | **Function** | **SIFT‡** | **PolyPhen 2⊱** |
| --- | --- | --- | --- | --- | --- | --- | --- |
| rs202018997 | exon1 | C>T | p.Ala64Thr | 0.0002 | missense | Deleterious | PROBABLY DAMAGING |
| rs201339654 | exon2 | G>A | p.Gly158Ser | NA | missense | Neutral | BENIGN |
|  |  | G>C | p.Gly158Arg | NA | missense | Neutral | BENIGN |
| rs61745930 | exon3 | A>G | p.Ile223Thr | 0.0104 | missense | Neutral | BENIGN |
| rs2296651 | exon4 | G>A | p.Ser267Phe | 0.0144 | missense | Deleterious | PROBABLY DAMAGING |
| rs759531965 | exon4 | C>T | p.Val263Ile | NA | missense | Neutral | BENIGN |

†MAF data from 1000 Genome (https://www.ncbi.nlm.nih.gov/variation/tools/1000genomes/). ‡SIFT (http://provean.jcvi.org/index.php) was using to predict the functional consequences of single nucleotide polymorphisms. Prediction outcome can be Neutral or Deleterious. ⊱Polyphen 2 (http://genetics.bwh.harvard.edu/pph2/) was using to predict the functional consequences of single nucleotide polymorphisms. Prediction outcome can be one of probably damaging, possibly damaging, or benign.

Table S2. The primers information used for the amplification of NTCP SNPs

| **SNP ID** | **Parameter** | **Strand(5’→3’)** |
| --- | --- | --- |
| rs202018997 | Forward | GAAGACCTTGCCCAGCACAAAG |
|  | Reverse | GTCTGCCCCATTCAACTTCACC |
| rs201339654 | Forward | TATTGTGGCCGTTTGGATTTGA |
|  | Reverse | ATGATGGGGACCTGAAGGACAA |
| rs61745930 | Forward | AACCCAGCAGAAAGCCAATAAAA |
|  | Reverse | GCTTCCCCACCTCTGTTCCTCT |
| rs2296651_ rs759531965 | Forward | TTGTTGGCAGGCTCAGGTCTAA |
|  | Reverse | CTTTGGTAGCAGCACTGGGACA |

Table S3. NTCP p.Ser267Phe variant and risk for overt HE

| **Model** | **Genotype** | **Non-HE**  **group** | **Overt HE**  **group** | **OR(95%CI)** | ***P* value** |
| --- | --- | --- | --- | --- | --- |
| Allele | G | 654(98.79) | 394(96.57) | 2.91(1.21-6.99) | **0.01** |
|  | A | 8(1.21) | 14(3.43) |  |  |
| Codominant | G/G | 323(97.58) | 192(94.11) | - | NA |
|  | G/A | 8(2.42) | 10(4.90) | NA |  |
|  | A/A | 0(0) | 2(0.98) |  |  |
| Dominant | G/G | 323(97.58) | 192(94.12) | 2.40(0.95-6.03) | 0.063 |
|  | G/A+A/A | 8(2.42) | 12(5.88) |  |  |
| Recessive | G/G+G/A | 331(100) | 202(99.02) | NA | NA |
|  | A/A | 0(0) | 2(0.98) |  |  |
| Additive | - | - | - | 2.50(1.050-5.70) | **0.038** |

Table S4. NTCP p.Ser267Phe variant and risk for HCC in cirrhosis

| **Model** | **Genotype** | **Cir without HCC**  **group** | **Cir with HCC**  **group** | **OR(95%CI)** | ***P* value** |
| --- | --- | --- | --- | --- | --- |
| Allele | G | 1456(96.81) | 563(96.74) | 1.02(0.60-1.76) | 0.93 |
|  | A | 48(3.19) | 19(3.26) |  |  |
| Codominant | G/G | 705(93.75) | 272(93.47) | - | NA |
|  | G/A | 46(6.12) | 19(6.52) | NA |  |
|  | A/A | 1(0.13) | 0(0) |  |  |
| Dominant | G/G | 705(93.75) | 272(93.47) | 1.10(0.58-2.08) | 0.77 |
|  | G/A+A/A | 47(6.25) | 19(6.52) |  |  |
| Recessive | G/G+G/A | 751(99.87) | 291(100) | NA | 1 |
|  | A/A | 1(0.13) | 0(0) |  |  |
| Additive | - | - | - | 1.06(0.57-1.97) | 0.86 |

Table S5. Association between NTCP p.Ser267Phe variant

and clinical parameters

| **laboratory tests** | **Model** | **Beta** | **SE** | **L95** | **U95** | **STAT** | ***P*-value** |
| --- | --- | --- | --- | --- | --- | --- | --- |
| AST | Additive | -114.6 | 30.25 | -173.9 | -55.28 | -3.788 | **1.55×10^-4^** |
| ALT | Additive | -129.4 | 35.87 | -199.7 | -59.09 | -3.607 | **3.14×10^-4^** |
| lgHBVDNA | Additive | 0.2316 | 0.1421 | -0.04698 | 0.5102 | 1.629 | 0.10 |
| HBeAg status | Additive | 0.04255 | 0.03031 | -0.01687 | 0.102 | 1.404 | 0.16 |
| AST | Dominant | -120.2 | 31.29 | -181.5 | -58.89 | -3.842 | **1.25×-10^-4^** |
| ALT | Dominant | -135.2 | 37.04 | -207.8 | -62.55 | -3.648 | **2.68×-10^-4^** |
| lgHBVDNA | Dominant | 0.2583 | 0.1458 | -0.02745 | 0.5441 | 1.772 | 0.08 |
| HBeAg status | Dominant | 0.04243 | 0.03136 | -0.01903 | 0.1039 | 1.353 | 0.18 |
| AST | HET† | -120.5 | 31.62 | -182.5 | -58.56 | -3.812 | **1.41×-10^-4^** |
| ALT | HET | -135.2 | 37.41 | -208.5 | -61.86 | -3.613 | **3.07×-10^-4^** |
| lgHBVDNA | HET | 0.2756 | 0.1469 | -0.01238 | 0.5636 | 1.876 | 0.06 |
| HBeAg status | HET | 0.04047 | 0.03168 | -0.02163 | 0.1026 | 1.277 | 0.20 |
| lgHBVDNA | HOM‡ | -0.7486 | 1.065 | -2.835 | 1.338 | -0.7031 | 0.48 |
| HBeAg status | HOM | 0.1278 | 0.1984 | -0.2611 | 0.5167 | 0.644 | 0.52 |
| ALT | HOM | -133.5 | 240.8 | -605.5 | 338.5 | -0.5544 | 0.58 |
| AST | HOM | -107 | 198 | -495.1 | 281.2 | -0.5401 | 0.59 |
| lgHBVDNA | Recessive | -0.7764 | 1.065 | -2.864 | 1.311 | -0.729 | 0.47 |
| HBeAg status | Recessive | 0.1237 | 0.1984 | -0.2651 | 0.5126 | 0.6237 | 0.53 |
| ALT | Recessive | -120.8 | 241.3 | -593.6 | 352.1 | -0.5006 | 0.62 |
| AST | Recessive | -94.95 | 198.5 | -484 | 294.1 | -0.4784 | 0.63 |

Abbreviations: Beta, Regression coefficient. SE, Standard Deviation. L95, Lower of the 95%CI. U95, Upper of the 95%CI. †HET is the GA genotype and ‡HOM is the AA genotype, which were compared with GG genotype. HET/HOM is the codominant model.

Table S6. Demographic and clinical features of patients enrolled

in the HBV sequences analysis

| **Characteristic** | **ALL**  **N=83** | **NTCP WT of HBV genoype B**  **N=39** | **NTCP S267 of HBV genoype B**  **N=24** | **NTCP WT of HBV genoype C**  **N=11** | **NTCP S267 of HBV genoype C**  **N=9** |
| --- | --- | --- | --- | --- | --- |
| gender, Male (%) | 56(67.5%) | 26(66.7%) | 18(75.0%) | 8(72.7%) | 4(44.4%) |
| Age,year,mean±SD | 32.47±1.14 | 32.64±1.46 | 35.46±2.80 | 29.09±2.39 | 27.89±1.67 |
| HBV DNA（lgIU/mL), mean±SD | 6.98±0.14 | 6.84±0.23 | 7.00±0.26 | 7.30±0.28 | 7.08±0.29 |
| ALT(IU/L), mean±SD | 47.73±3.06 | 51.53±5.57 | 40.75±4.22 | 45.45±4.45 | 52.55±7.14 |
| AST(IU/L), mean±SD | 35.42±1.72 | 38.15±2.87 | 31.29±2.55 | 36.82±4.85 | 32.89±3.77 |
| HBeAg positive （%） | 63(75.9%) | 29(74.4%) | 17(70.8%) | 9(81.8%) | 8(88.9%) |

Table S7. Demographic and clinical features of individuals

with homozygous p.Ser267Phe mutation

| **Patient ID** | **Sex** | **Age（years）** | **Diagnosis** | **HBV DNA（IU/mL)** | **HBsAg** | **HBeAg** | **HBeAb** | **Total bile acid**  **(umol/L)** |
| --- | --- | --- | --- | --- | --- | --- | --- | --- |
| 1 | M | 23 | CHB | -^†^ | +^‡^ | - | + | 110 |
| 2 | M | 22 | CHB | - | + | - | + | 17.2 |
| 3 | M | 61 | CIR | - | + | - | + | 59.9 |
| 4 | M | 25 | ACLF | 691 | + | + | - | 65.6 |
| 5 | M | 69 | ACLF | 1.55E5 | + | - | + | 216.1 |
| 6 | M | 50 | CHB | 112 | + | - | + | 109 |

†“-”negative； ‡“+”positive
